# Effects of Exercise Interventions on Inflammatory Parameters in Acutely Hospitalized Older Patients: A Systematic Review and Meta-Analysis of Randomized Controlled Trials

**DOI:** 10.3390/jcm10020290

**Published:** 2021-01-14

**Authors:** Robinson Ramírez-Vélez, Antonio García-Hermoso, Nicolás Martínez-Velilla, Fabricio Zambom-Ferraresi, Mikel L. Sáez de Asteasu, Anel E. Recarey, Mikel Izquierdo

**Affiliations:** 1 Department of Health Sciences, Public University of Navarra, 31008 Pamplona, Spain; robin640@hotmail.com (R.R.-V.); antonio.garcia.h@usach.cl (A.G.-H.); nicolas.martinez.velilla@navarra.es (N.M.-V.); fabriciogigante@hotmail.com (F.Z.-F.); mikel.lopez.saezdeasteasu@gmail.com (M.L.S.d.A.); anel1115.recarey@gmail.com (A.E.R.); 2 Centro de Investigación Biomédica en Red de Fragilidad y Envejecimiento Saludable (CIBERFES), Instituto de Salud Carlos III, 28029 Madrid, Spain; 3 Laboratorio de Ciencias de la Actividad Física, el Deporte y la Salud, Facultad de Ciencias Médicas, Universidad Santiago de Chile, 7500618 Santiago, Chile

**Keywords:** physical activity, aging, inflammation, hospitalization, elderly

## Abstract

The purpose of this systematic review and meta-analysis was to appraise the acute effects of exercise training on inflammatory parameters in hospitalized older adults. We conducted a systematic review using the Preferred Reporting Items for Systematic Reviews and Meta-Analyses guidelines. Web of Science, Medline and PubMed were searched for studies published until August 2020. The review included all randomized controlled trials (RCTs) that evaluated and compared the effect of exercise versus usual care on inflammatory parameters in acutely hospitalized older adults. Two reviewers independently assessed the studies. The quality of all the included studies was assessed using the DerSimonian–Laird random-effects inverse-variance model. Five studies (275 participants) met the inclusion criteria. The exercise interventions included resistance or multicomponent intervention programs. The results indicate that, compared with usual care, exercise interventions have a positive impact on overall inflammatory parameters, including C-reactive protein (CRP) and insulin-like growth factor-I (IGF-1) (Hedge’s *g* = −0.19, 95% confidence interval [CI] −0.33 to −0.04, *p* = 0.011, *I*^2^ = 0%). However, analyses of individual inflammatory parameters revealed a non-significant trend for reductions in CRP (Hedge’s *g* = −0.20, 95% CI −0.47 to 0.07, *p* = 0.151, *I*^2^ = 31.2%) and IGF-I (Hedge’s *g* = −0.34, 95% CI −0.79 to 0.11, *p* = 0.138, *I*^2^ = 0%). On the basis of this review, we conclude that exercise during acute hospitalization offers a mild improvement in the inflammatory profile over usual care in older patients. Nevertheless, due to limited number of RCTs, our findings must be interpreted with caution and confirmed in future studies.

## 1. Introduction

Hospitalization can result in rapid deconditioning, overall weakness and functional decline [1], which is related to a substantial loss (~7–10%) in total lean leg mass in as little as 7 days of in-hospital inactivity [2,3]. Loss of muscle mass in hospitalized older patients is associated with a lower likelihood of survival [2,4], and can cause disability even in healthy patients. Hospital-induced disability is—in turn—related to a loss of the ability to independently perform one or more activities of daily living from hospital admission to discharge in approximately one third of older patients, even in the case of successful treatment [5,6]. It is also associated with an increased risk of re-admission and mortality [5,6] and—in the case of critically ill patients—severe muscle wasting in the first week of hospitalization [5,7]. This is due largely to the combination of disease and low mobility levels provoked by prolonged periods of bed rest and/or forced physical inactivity [1,2,3,4,5,6,7].

Acute hospitalization per se frequently produces an inflammatory cascade in older populations, inducing a catabolic effect on skeletal muscle metabolism [8]. Additionally, the chronic low-grade inflammation associated with aging increases the vulnerability of older adults to the negative impact of hospitalization because of its relationship with changes in body composition and declining physical function [2,4].

Several non-pharmacological strategies have been shown to prevent the functional decline that often accompanies periods of forced physical inactivity, such as those imposed by hospitalization. Early exercise and/or mobilization interventions—from simple sit-to-stand and walking exercises to more complex resistance exercises—can stimulate the release of skeletal muscle-derived myokines, which are responsible for some of the beneficial effects of exercise in older adults, primarily by enhancing anti-inflammatory pathways [2,9]. Likewise, it has been hypothesized that exercise interventions performed by older adults during acute hospitalization elicit changes in protein content or function in blood, contributing to the numerous multi-system health benefits observed with continued training [10]. Thus, structured early exercise and rehabilitation programs can prevent muscle function deterioration, abbreviate the periods of exacerbation of acute illness, and reduce the impact of subsequent health crises in hospitalized older adults [9]. The potential for early exercise and mobilization interventions in hospitalized older adults to increase the anti-inflammatory and analgesic actions of medications and ameliorate inflammation and age-related diseases is a relatively new concept in health promotion research that is gaining traction as an important component of care for patients. Nevertheless, the acute effect of exercise on inflammatory parameters during the hospitalization of older adults remains undefined.

The purpose of this systematic review and meta-analysis was to analyze the acute effects of exercise training on inflammatory parameters in acutely hospitalized older adults. We hypothesized that the aforementioned interventions would favorably modulate inflammatory signaling during hospitalization compared to usual care, which might highlight new signaling factors that contribute to physiological adaptions.

## 2. Methods

The conduct and reporting of the present systematic review and meta-analysis conform to the Preferred Reporting Items for Systematic Reviews and Meta-Analyses (PRISMA) [11]. This review was not prospectively registered.

### 2.1. Eligibility Criteria

The included studies were eligible if they met the population, intervention, comparison, outcomes and study design (PICOS) criteria listed in Table 1. In brief, the included articles had to report exercise protocol and parameters, and the assessment of at least one outcome of an inflammatory parameter. Exclusion criteria were the following: (i) a single session of physical exercise or chronic experimental intervention (i.e., occurring also after acute hospitalization); (ii) evaluation of inflammatory parameters only as a basal assessment during the study; (iii) case-reports, case-series, single-case studies, dissertations, and conference proceedings.

### 2.2. Information Sources and Search

The review included all randomized controlled trials (RCTs) that evaluated the effect of exercise compared with usual care on inflammatory parameters in older adults during acute hospitalization. Queries of the literature were performed using the electronic databases Web of Science, Medline and PubMed for studies published in English or Spanish from the earliest date available (1975) until August 2020. The search terms employed through the scientific databases were made with a combination of subject headings and text words for older adults, inpatients, exercise, and usual care: [(“older adults” OR “older patients” OR elderly OR elders OR geriatric* OR eldest OR oldest OR senior OR octogenarian OR nonagenarian OR centenarian) AND (hospital* OR “acute care” OR inpatient) AND (physical activity OR exercise* OR “physical therapy” OR rehabilitation OR training OR mobilization OR ambulat*) AND (random* OR control* OR “usual care”)] AND (“inflammation” OR inflame). We narrowed down the search by including only RCTs. The complete search strategy is shown in Supplementary Material Table S1.

### 2.3. Study Selection and Data Collection Process

Potentially eligible studies identified by the search strategy were independently screened by two reviewers (A.G.-H. and R.R.-V.) through the evaluation of titles and abstracts. If an abstract did not provide enough information for evaluation based on the inclusion and exclusion criteria, the full article was retrieved for a full text assessment.

Regarding interventions, we recorded frequency (times per week and times per day), session duration, type of exercise and intensity, volume (number of repetitions, series, and exercises), and training place, to compare the similarity of training methods between trials.

### 2.4. Risk of Bias in Individual Studies

Two reviewers (A.G.-H. and R.R.-V.) independently assessed the methodological quality using the Physiotherapy Evidence Database (PEDro) scale [12], which is based on the Delphi List but modified by expert consensus to include two additional items (items 8 and 10). The Pedro scale scores 11 items: 1. eligibility criteria were specified; 2. subjects were randomly allocated to groups (in a crossover study, subjects were randomly allocated an order in which treatments were received); 3. allocation was concealed; 4. the groups were similar at baseline regarding the most important prognostic indicators; 5. there was blinding of all subjects; 6. there was blinding of all therapists who administered the therapy; 7. there was blinding of all assessors who measured at least one key outcome; 8. measures of at least one key outcome were obtained from more than 85% of the subjects initially allocated to groups; 9. all subjects for whom outcome measures were available received the treatment or control condition as allocated or, where this was not the case, data for at least one key outcome were analyzed by “intention to treat”; 10. the results of between-group statistical comparisons are reported for at least one key outcome; 11. the study provides both point measures and measures of variability for at least one key outcome. Each satisfied item (except the first item) contributes 1 point to the total PEDro score (range = 0–10 points). RCTs were considered of high quality when the PEDro score was ≥6.

### 2.5. Synthesis of Results

All analyses were conducted using the DerSimonian-Laird random-effects inverse-variance model using STATA software (version 16.1; StataCorp, College Station, TX, USA). Data were pooled only if outcomes were reported in at least two studies. Changes in both parameters in the RCTs were calculated by subtracting the differences between the exercise and control groups using the pooled standard deviation (SD) of change in both groups (Hedge’s g). If change score SDs were not available, they were calculated from 95% confidence intervals (CI) for either change outcome or exercise training effect differences, as well as pre- and post-SD values [13]. A subgroup analysis according to inflammatory parameters was also included for C-reactive protein (CRP) and insulin-like growth factor-I (IGF-1) data. Heterogeneity across RCTs was calculated using the inconsistency index (*I*^2^) [14], and Egger’s regression intercept test was used to detect publication bias. No sub-group analysis was performed due to the limited number of studies.

## 3. Results

### 3.1. Study Selection

**Figure 1:**
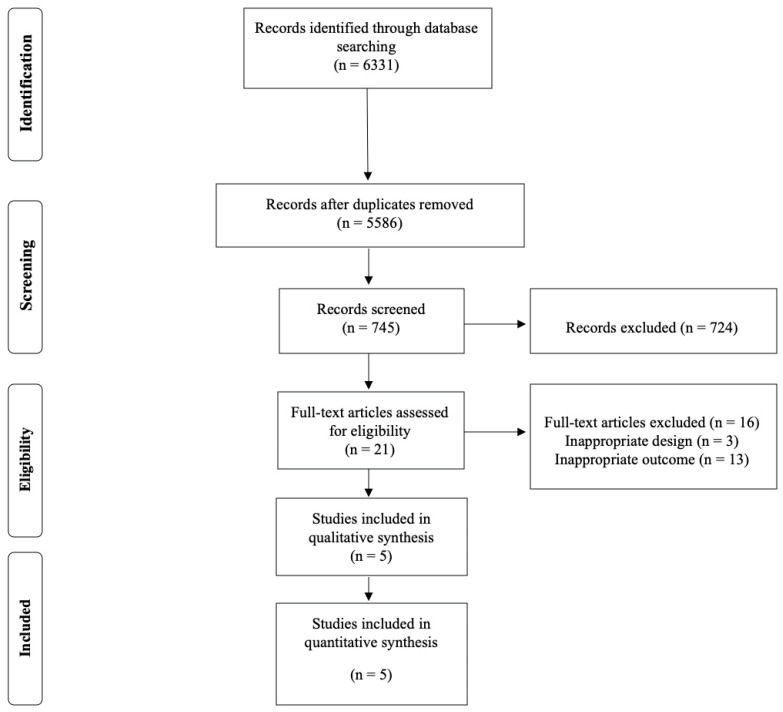
Flowchart of literature search.

A total of five RCTs were included in the meta-analysis [2,15,16,17,18]. The PRISMA flow diagram is shown in Figure 1.

### 3.2. Study Characteristics

A summary of the five studies included in this study is detailed in Table 2. The studies involved a total of 275 participants (mean age of 78.8 years), ranging from 36 [18] to 76 [15] older adults per study. All RCTs included males and females (64.8% and 35.2%, respectively).

The studies were conducted at hospitals in France [15], Belgium [18], Brazil [16] and Spain [2,17]. Three studies included patients with chronic obstructive pulmonary disease [16,17,18] and two studies included patients with general medical conditions [2,15]. Exercise interventions included resistance [15,16,18] or multicomponent [2,17] intervention programs.

### 3.3. Risk of Bias within Studies

All included RCTs used a random allocation between groups and provided points and estimates of variability. Only two studies used a concealed allocation [16,18]. Blinding of participants and therapists was not possible because of the nature of the exercise interventions. Assessor blinding was employed in three of the RCTs [2,16,17]. Four studies could be considered as high quality. Details are described in Table 3.

### 3.4. Synthesis of Results

**Figure 2:**
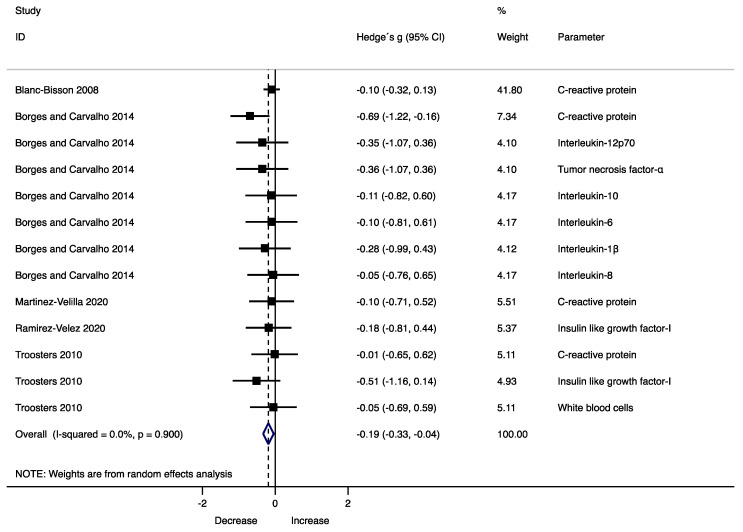
Forest plot showing the effect size (Hedges’ g) of physical exercise programs on overall inflammatory profile.

The results indicate that exercise significantly decreased overall inflammation as compared with usual care (Hedge’s *g* = −0.19, 95% CI −0.33 to −0.04, *p* = 0.011, *I*^2^ = 0%) (Figure 2). However, analyses of individual inflammatory parameters showed a non-significant trend for reductions in CRP (Hedge’s *g* = −0.20, 95% CI −0.47 to 0.07, *p* = 0.151, *I*^2^ = 31.2%) and IGF-I (Hedge’s *g* = −0.34, 95% CI −0.79 to 0.11, *p* = 0.138, *I*^2^ = 0%) (Supplementary Material Figure S1).

Regarding publication bias, the results show a *p*-value of 0.297, reflecting no publication bias for the present meta-analysis (i.e., no small-study effects).

## 4. Discussion

The main aim of this systematic review and meta-analysis was to appraise the effects of exercise training, mainly resistance protocols, on inflammatory parameters in acutely hospitalized older adults. Although our findings must be interpreted carefully due to the limited number of RCTs assessed, results indicate that exercise during acute hospitalization could offer a mild improvement in the inflammatory status as compared with usual care. Therefore, physical exercise during acute hospitalization could decrease the vulnerability of older adults to risks normally incurred during their hospital stay [2,4].

Several studies have confirmed that physical exercise is an effective treatment modality to improve exercise tolerance, reduce morbimortality, and enhance functional and cognitive capacity in older adults during acute care hospitalization, thus improving their quality of life during and following this period [2,9,17,20,21,22]. However, there is a paucity of studies exploring the physiological mechanisms that control these changes.

Chronic systemic inflammation is characterized by a dysregulated immune response that fails to resolve naturally, which exacerbates the mobilization of defense components to create a long-term unresolved immune response [23]. Chronic systemic inflammation is related to impaired physical function, morbidity and mortality in the elderly and is implicated in several age-related conditions, such as frailty and sarcopenia syndrome, diseases associated with cognitive impairment such as Alzheimer’s disease, diseases associated with cardiometabolic impairment such as atherosclerosis, hypertension and diabetes, and also with cancer [24,25,26,27,28,29,30,31]. Physical exercise is hypothesized to reduce age-related inflammation through the stimulation of various physiological mechanisms—thus improving the quality of life and general health of the elderly [17,26]. In this regard, previous studies have assessed the effects of different types of exercise training on inflammation in healthy older adults, older adults with specific diseases or conditions, and in frail older adults, showing its benefits. For example, in a systematic review of 34 studies, Liberman et al., 2017 [27] concluded that different types of exercise intervention have variable effects on inflammation, principally by lowering the circulating levels of the biomarkers CRP, IL-6 and other inflammatory proteins, which appear to mediate some of the metabolic effects of exercise.

The studies included in the present review [2,15,16,17,18] assessed the effects of resistance or multicomponent exercise intervention on circulating proteins associated with inflammation and its processes, including CRP [15,16,17,18], IGF-I [2,18] and IL-6 [16]. CRP is an acute-phase hepatic protein, the circulating levels of which increase in response to inflammation. Specifically, CRP is released in response to IL-6 secretion by macrophages, T-cells and/or adipocytes, and its principal role is to bind to the surface of dead or dying cells (and some types of bacteria) to activate the complement system and facilitate phagocytosis by macrophages. IL-6 is often considered as a pro-inflammatory cytokine, stimulating inflammatory and auto-immune processes related to multiple diseases such as diabetes, atherosclerosis, Alzheimer’s disease and cancer [28,29]. However, it can also act as an anti-inflammatory myokine by inhibiting the effects of TNF-α and IL-1, and stimulating the production of IL-1 receptor antagonist and IL-10 [30,31,32]. IGF-1 is a hormone similar in molecular structure to insulin that plays an important role in childhood growth, and has anabolic effects in adults. Nonetheless, when produced in excess, IGF-1 is associated with subclinical inflammation by promoting systemic inflammation, probably via the MAPK signaling pathway [33].

The limited scientific data that are currently available prevent the establishment of definitive conclusions on the effect of exercise interventions on inflammation outcomes in acutely hospitalized older adults. Nonetheless, the results of this review should help to establish focal points and opportunities for the design of future research, aiding practitioners and researchers in the design and implementation of exercise interventions in acutely hospitalized older adults, which are warranted before definitive conclusions can be drawn on its effect on these complex and variable parameters.

## 5. Conclusions

We found significant anti-inflammatory effects of resistance and/or multicomponent exercise in acutely hospitalized older adults. There is a clinical consensus that immobility among hospitalized older adults, combined with the natural disease process of incapacitation, aging and other aspects of inpatient settings, induces adverse outcomes. The results from this meta-analysis suggest that exercise interventions, mainly resistance protocols, appear to be feasible and safe for older patients and may have a positive effect in reducing inflammation compared with usual care. In this context, although the reduction observed in the present study was small, this result could be considered clinically relevant, since inflammation plays a very important role in various physiological pathways and contributes to the process of incapacitation and death. This may help to clarify how exercise interventions provoke the improvement of functional capacity, quality of life, and other health outcomes that are associated with inflammation in older adults during acute care hospitalization. However, due to the limited number of RCTs, our findings should be interpreted with caution, and more studies are needed to draw definite conclusions on this topic. Furthermore, future recommendations include the realization of standardized, well-designed RCTs to study the effect of resistance and/or multicomponent exercise interventions on inflammatory outcomes in hospitalized older adults.

## Figures and Tables

**Table 1:** Inclusion criteria.

Design:
Randomized controlled trials (RCTs)
Parallel and crossover design trials were included
Published in English or Spanish
**Participants:**
Older adults (mean age ≥65 years)
**Intervention:**
Exercise training
**Outcome measures:**
Inflammatory parameters
**Comparisons:**
Exercise versus usual care or attention (control), based on a traditional parallel RCTs design
The same group of subjects performing different interventions versus no intervention, based on a crossover RCTs design

**Table 2:** Summary of included studies.

Author, Year, Country	Sample (% Female)/Age (Range or Mean)	Type	Intervention Length (Days)	Frequency (Sessions/Day)	Duration (min)	Inflammatory Parameters
Blanc-Bisson et al., 2008 [15]; France	76 (28%)/85.4 years old	Resistance	5	2	30	C-reactive protein
Borges and Carvalho., 2014 [16]; Brazil	39 (37.9%)/65.9 years old	Resistance	3	1	-	IL-6; IL-8; IL-10; IL12p-70; IL-1β, Tumor necrosis factor-α; C-reactive protein
Martínez-Velilla et al., 2020 [17]; Spain	86 (43%)/87 years old	Multicomponent	5–7	2	20	C-reactive protein
Ramírez-Vélez et al., 2020 [19]; Spain	38 (42.1%)/87.9 years old	Multicomponent	5–7	2	20	Insulin like growth factor-I
Troosters et al., 2010 [18]; Belgium	36 (25%)/68 years old	Resistance	7	1	-	C-reactive protein; White blood cells neutrophil count; Insulin like growth factor-I

IL, interleukin.

**Table 3:** Assessment risk of bias (PEDro scale).

Study, Year	1 *	2	3	4	5	6	7	8	9	10	11	Total
Blanc-Bisson et al.; (2008) [15]	1	1	0	1	0	0	0	0	1	1	1	5
Borges and Carvalho.; (2014) [16]	0	1	1	1	0	0	1	0	0	1	1	6
Martínez-Velilla et al.; (2020) [17]	1	1	0	1	0	0	1	0	1	1	1	6
Ramírez-Vélez et al.; (2020) [19]	1	1	0	1	0	0	1	0	1	1	1	6
Troosters et al.; (2010) [18]	1	1	1	1	0	0	0	1	0	1	1	6

* Item 1 does not contribute to the total score.

## Data Availability

Not applicable.

## Supplementary Materials

The following are available online at https://www.mdpi.com/2077-0383/10/2/290/s1. Table S1: PubMed search strategy; Figure S1: Forest plot showing the effect size (Hedges’ g) of physical exercise programs on inflammatory parameters.
