# Supplementary material for: Effects of Exercise Interventions on Inflammatory Parameters in Acutely Hospitalized Older Patients: A Systematic Review and Meta-Analysis of Randomized Controlled Trials
Source: J Clin Med. 2021 Jan 14;10(2):290. doi: 10.3390/jcm10020290 (PMC7830051; doi:10.3390/jcm10020290)

Table S1. PubMed search strategy.

**PubMed**
(((("aged"[MeSH Terms] OR "aged"[All Fields] OR ("older"[All Fields] AND "adults"[All Fields]) OR "older adults"[All Fields] OR (("older"[All Fields] OR "olders"[All Fields]) AND ("patient s"[All Fields] OR "patients"[MeSH Terms] OR "patients"[All Fields] OR "patient"[All Fields] OR "patients s"[All Fields])) OR ("aged"[MeSH Terms] OR "aged"[All Fields] OR "elderly"[All Fields] OR "elderlies"[All Fields] OR "elderly s"[All Fields] OR "elderlys"[All Fields]) OR ("elder s"[All Fields] OR "elders"[All Fields] OR "sambucus"[MeSH Terms] OR "sambucus"[All Fields] OR "elder"[All Fields]) OR "geriatric*"[All Fields] OR "eldest"[All Fields] OR "oldest"[All Fields] OR ("senior"[All Fields] OR "seniorities"[All Fields] OR "seniority"[All Fields] OR "seniors"[All Fields]) OR ("aged, 80 and over"[MeSH Terms] OR "80 and over aged"[All Fields] OR "octogenarian"[All Fields] OR "octogenarians"[All Fields]) OR ("aged, 80 and over"[MeSH Terms] OR "80 and over aged"[All Fields] OR "centenarian"[All Fields] OR "centenarians"[All Fields])) AND ("hospital s"[All Fields] OR "hospitalisation"[All Fields] OR "hospitalization"[MeSH Terms] OR "hospitalization"[All Fields] OR "hospitalised"[All Fields] OR "hospitalising"[All Fields] OR "hospitality"[All Fields] OR "hospitalisations"[All Fields] OR "hospitalizations"[All Fields] OR "hospitalize"[All Fields] OR "hospitalized"[All Fields] OR "hospitalizing"[All Fields] OR "hospitals"[MeSH Terms] OR "hospitals"[All Fields] OR "hospital"[All Fields])) OR ("acute care"[Journal] OR ("acute"[All Fields] AND "care"[All Fields]) OR "acute care"[All Fields]) OR ("inpatient s"[All Fields] OR "inpatients"[MeSH Terms] OR "inpatients"[All Fields] OR "inpatient"[All Fields]) OR ("exercise"[MeSH Terms] OR "exercise"[All Fields] OR ("physical"[All Fields] AND "activity"[All Fields]) OR "physical activity"[All Fields]) OR ("education"[MeSH Subheading] OR "education"[All Fields] OR "training"[All Fields] OR "education"[MeSH Terms] OR "train"[All Fields] OR "train s"[All Fields] OR "trained"[All Fields] OR "training s"[All Fields] OR "trainings"[All Fields] OR "trains"[All Fields]) OR ("mobilisation"[All Fields] OR "mobilisations"[All Fields] OR "mobilise"[All Fields] OR "mobilised"[All Fields] OR "mobiliser"[All Fields] OR "mobilisers"[All Fields] OR "mobilises"[All Fields] OR "mobilising"[All Fields] OR "mobilization"[All Fields] OR "mobilizations"[All Fields] OR "mobilize"[All Fields] OR "mobilized"[All Fields] OR "mobilizer"[All Fields] OR "mobilizers"[All Fields] OR "mobilizes"[All Fields] OR "mobilizing"[All Fields]) OR "ambulat*"[All Fields] OR "random*"[All Fields] OR "control*"[All Fields] OR ("usual"[All Fields] AND "care"[All Fields])) AND ("inflammation"[MeSH Terms] OR "inflammation"[All Fields] OR "inflammations"[All Fields] OR "inflammation s"[All Fields])) OR "inflame"[All Fields]

Figure S1. Forest plot showing the effect size (Hedges' g) of physical exercise programs on inflammatory parameters.

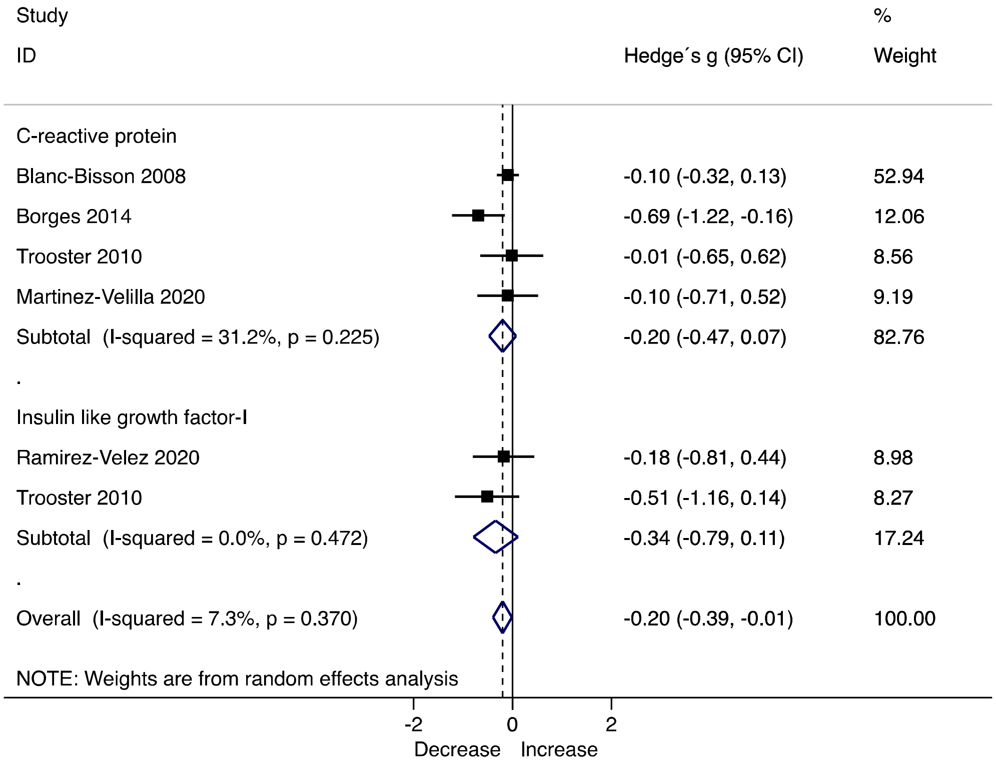

Supplement: Supplementary file 1 [file jcm-10-00290-s001.pdf]
